# Variability in the *sxt* Gene Clusters of PSP Toxin Producing *Aphanizomenon gracile* Strains from Norway, Spain, Germany and North America

**DOI:** 10.1371/journal.pone.0167552

**Published:** 2016-12-01

**Authors:** Andreas Ballot, Leonardo Cerasino, Vladyslava Hostyeva, Samuel Cirés

**Affiliations:** 1 Norwegian Institute for Water Research, Oslo, Norway; 2 IASMA Research and Innovation Centre, Istituto Agrario di S. Michele all'Adige - Fondazione E. Mach, S. Michele all’Adige (Trento), Italy; 3 Departamento de Biología, Universidad Autónoma de Madrid, Madrid, Spain; University of New South Wales, AUSTRALIA

## Abstract

Paralytic shellfish poisoning (PSP) toxin production has been detected worldwide in the cyanobacterial genera *Anabaena*, *Lyngbya*, *Scytonema*, *Cuspidothrix* and *Aphanizomenon*. In Europe *Aphanizomenon gracile* and *Cuspidothrix issatschenkoi* are the only known producers of PSP toxins and are found in Southwest and Central European freshwater bodies. In this study the PSP toxin producing *Aphanizomenon* sp. strain NIVA-CYA 851 was isolated from the Norwegian Lake Hillestadvannet. In a polyphasic approach NIVA-CYA 851 was morphologically and phylogenetically classified, and investigated for toxin production. The strain NIVA-CYA 851 was identified as *A*. *gracile* using 16S rRNA gene phylogeny and was confirmed to produce neosaxitoxin, saxitoxin and gonyautoxin 5 by LC-MS. The whole *sxt* gene clusters (circa 27.3 kb) of four *A*. *gracile* strains: NIVA-CYA 851 (Norway); NIVA-CYA 655 & NIVA-CYA 676 (Germany); and UAM 529 (Spain), all from latitudes between 40° and 59° North were sequenced and compared with the *sxt* gene cluster of reference strain *A*. *gracile* NH-5 from the USA. All five *sxt* gene clusters are highly conserved with similarities exceeding 99.4%, but they differ slightly in the number and presence of single nucleotide polymorphisms (SNPs) and insertions/deletions (In/Dels). Altogether 178 variable sites (44 SNPs and 4 In/Dels, comprising 134 nucleotides) were found in the *sxt* gene clusters of the Norwegian, German and Spanish strains compared to the reference strain. Thirty-nine SNPs were located in 16 of the 27 coding regions. The *sxt* gene clusters of NIVA-CYA 851, NIVA-CYA 655, NIVA-CYA 676 and UAM 529, were characterized by 15, 16, 19 and 23 SNPs respectively. Only the Norwegian strain NIVA-CYA 851 possessed an insertion of 126 base pairs (bp) in the noncoding area between the *sxtA* and *sxtE* genes and a deletion of 6 nucleotides in the *sxtN* gene. The *sxtI* gene showed the highest variability and is recommended as the best genetic marker for further phylogenetic studies of the *sxt* gene cluster of *A*. *gracile*.

This study confirms for the first time the role of *A*. *gracile* as a PSP toxin producer in Norwegian waters, representing the northernmost occurrence of PSP toxin producing *A*. *gracile* in Europe known so far.

## Introduction

Cyanobacteria belonging to the order Nostocales and Oscillatoriales have been identified worldwide as producers of neurotoxic paralytic shellfish poisoning toxins (PSP toxins) or saxitoxins [1–4]. Confirmed producers of PSP toxins are *Aphanizomenon gracile*, *Cuspidothrix issatschenkoi* (formerly *Aphanizomenon issatschenkoi*), *Cylindrospermopsis raciborskii*, *Cylindrospermum stagnale*, *Dolichospermum circinale* (formerly *Anabaena circinalis*), *Geitlerinema amphibium*, *Geitlerinema lemmermannii*, *Lyngbya wollei*, *Phormidium uncinatum*, *Raphidiopsis brookii* and *Scytonema* cf. *crispum*, reported in Australia, Brazil, Germany, New Zealand, North America, Portugal and Spain, respectively [2, 4–10]. In central and southern Europe, the only known producers of PSP toxins to date are *A*. *gracile* and *Cu*. *issatschenkoi* [3, 10–13]. PSP toxins have also been confirmed in cyanobacterial blooms in northern Europe (Denmark and Finland) and Eastern Europe (Greece) but without unambiguous identification of the producing organism [14–17].

*A*. *gracile* strain NH-5 was isolated from a pond in New Hampshire (USA) in 1980 [18]. At that time the strain was identified as *Aphanizomenon flos-aquae* but later revised to *Aphanizomenon gracile* NH-5 using genetic methods [10, 18, 19]. It is one of the five cyanobacterial PSP toxin producing species of which the whole putative PSP toxin encoding gene cluster (*sxt* gene cluster) has been identified [20, 21]. With a size of around 27.5 kb the *sxt* gene cluster in *A*. *gracile* strain NH-5 is the second smallest of the *sxt* gene clusters found in *A*. *gracile*, *D*. *circinale*, *C*. *raciborskii*, *R*. *brookii* and *L*. *wollei* [20–23]. The *sxt* gene cluster of *A*. *gracile* strain NH-5 is more similar in gene content and cluster organization to that of *D*. *circinale* than to that of *C*. *raciborskii*, *R*.*brookii* or *L*. *wollei* [20–23]. Common to all PSP toxin producers is a set of 14 core genes (e.g. *sxtA*, *sxtB*, *sxtD*, *sxtG*, *sxtS*, *sxtU*, *sxtV*, *sxtW*, *sxtH*, *sxtT*, *sxtI*, *sxtJ*, *sxtK*) while the composition of the other genes in the *sxt* gene clusters varies between species [20, 22–24]. The putative functions of the core genes include among others the role of acyl-carrier protein (ACP), dioxygenase reductase or electron carrier methylation. They are also involved in claisen condensation, cyclisation, desaturation, amidinotransfer, C1 reduction, C12 hydroxylation and carbamoylation [25].

Tailoring genes (e.g. *sxtC*, *sxtL*, *sxtN*, *sxtX*) are responsible for e.g. decarbamoylation, N-sulfotransfer, and N1-hydroxylation. Other genes in the *sxt* gene cluster are auxiliary genes (e.g. *sxtM*, *sxtPer*), responsible for export or regulation. Genes with an unknown function are e.g. *sxtE*, *sxtP*, *sxtQ* and *sxtR* [25]. To date, the comparison of whole *sxt* gene clusters has been conducted on an interspecific level for only one strain each of *D*. *circinale*, *A*. *gracile* NH-5, *C*. *raciborskii* T3, *Raphidiopsis brookii* D9 and *L*. *wollei* [20–23]. No comparisons have been performed on an intraspecific level.

The known PSP toxin producers are characterized by varying toxin profiles. Some *A*. *gracile* strains mainly produce neosaxitoxin (NEO), saxitoxin (STX) and gonyautoxin5 (GTX5) while other strains produce only STX and decarbamoylsaxitoxin (dcSTX) or NEO and STX [3, 11–13, 18]. In extracts of *D*. *circinale* STX, GTX2/3, N-sulfocarbamoyl toxins C1/2, dcSTX and decarbamoylgonyautoxin2/3 (dcGTX2/3) have been identified while *C*. *raciborskii* produces NEO, dcNEO, dcSTX & STX and *R*. *brookii* produces GTX2/3, STX & low amounts of dcGTX2/3 [23, 26, 27]. The most unique PSP toxin profile is described from *L*. *wollei*. which produces *L*. *wollei* toxins (LWT) 1–6 not found in other cyanobacteria to date, as well as dcGTX2 and dcGTX3 [28].

In Europe, confirmed PSP toxin producing *A*. *gracile* strains have been isolated from the following locations: two Northeast German lakes Scharmützelsee (52° 14'51" N, 14°03'17" E) and Melangsee (52° 09'41" N, 13°59'19" E), the Portuguese Lake Crato (coordinates not known), the French reservoir Champs sur Marne (48°51'50" N, 2°35'53" E) and the Rosarito reservoir (40°06' N, 5°18' W) in Spain [3, 11–13, 29]). The sixteen *A*. *gracile* strains from Germany and Spain and the *A*. *gracile* strain NH-5 from North America form monospecific and highly supported clusters for *sxt* genes (*sxtA*, *sxtG*, *sxtI*, *sxtH* and *sxtX*) [3, 29]. Variable nucleotide sequences have been found in partial sequences of the *sxtH* and *sxtI* genes [29].

In July 2013 a bloom of *Aphanizomenon* spp. was observed in Norwegian L. Hillestadvannet and a putative PSP toxin producing *Aphanizomenon* cf. *gracile* strain was isolated and cultured as strain NIVA-CYA 851 in the algal culture collection of the Norwegian Institute for Water Research (NIVACCA). A preliminary test using ELISA (enzyme-linked immunosorbent assay) confirmed the presence of PSP toxins in this culture. In Norwegian water bodies, *A*. *gracile* has been rarely observed and PSP toxins have not yet been detected in Norway. The aim of this study was therefore to identify the isolated Norwegian strain using a polyphasic approach. Furthermore, the potential as a PSP toxin producer was investigated chemically and genetically. The complete *sxt* gene cluster was analyzed and compared to the *sxt* gene cluster available from *A*. *gracile* strain NH-5. Additionally, the *sxt* gene clusters of three PSP toxin producing *A*. *gracile* strains (NIVA-CYA 655, NIVA-CYA 676 and UAM 529) from Germany and Spain were investigated for comparison. These analyses increase our knowledge of the distribution of PSP producing *A*. *gracile*, their phylogenetic relationship and the intraspecific variations in toxin gene clusters

## Material and Methods

### Isolation and selection of strains

Using a microcapillary, single putative *A*. cf. *gracile* filaments were isolated from a phytoplankton sample from the Norwegian L. Hillestadvannet (59° 31'42.69" N, 10° 10'11.89" E) taken in August 2013. They were washed five times and placed in wells on a microtiter plate containing 300 μL Z8 medium [30]. After successful growth, one strain was placed in a 50mL Erlenmeyer flask containing 20mL Z8 medium and maintained at 22°C. The strain was classified on the basis of morphological traits according to Komárek [31]. Morphological studies were conducted using a Leica DM2500 light microscope, Leica DFC450 camera and Leica Application Suite software (LAS) (Leica, Oslo, Norway). The strain used in this study is maintained with the number NIVA-CYA 851 in the culture collection of algae at the Norwegian Institute for Water Research, Oslo, Norway.

For genetic comparisons *sxt* gene clusters of *A*. *gracile* strains NIVA-CYA 655 (AB2008/16) and NIVA-CYA 676 (AB2008/48) isolated from the German lakes Scharmützelsee and Melangsee, respectively, and the strain UAM 529 isolated from Spanish Rosarito reservoir were also analyzed [3, 13]. The 16S rRNA genes sequences of these strains were obtained from GenBank.

### Genomic DNA extraction, PCR amplification and sequencing

A modified isolation of genomic DNA was conducted after Ballot et al. [32]. Instead of horizontal vortexing, a bead beating step (3 × 30 sec, 6700 rpm) in a Precellys 24 Beadbeater (Bertin, Technologies, Saint Quentin, France), was used to disrupt the cells.

PCRs for 16S rRNA gene and parts of the *sxt* gene cluster were performed on a Bio-Rad CFX96 Real-Time PCR Detection System (Bio-Rad Laboratories, Oslo, Norway) using the iProof High-Fidelity PCR Kit (Bio-Rad Laboratories, Oslo, Norway). 16S rRNA gene and s*xt* cluster fragments were amplified in separate PCR reactions using the same cycling protocol comprising of: one cycle of 5 min at 94°C, and then 35 cycles each consisting of 10 s at 94°C, 20 s at 62°C, and 20 s at 72°C, followed by a final elongation step of 72°C for 5 min. PCR products were visualized by 1.5% agarose gel electrophoresis with GelRed staining and UV illumination. Amplified PCR products were purified through QIAquick PCR purification columns (QIAGEN, Hilden, Germany), and the DNA was eluted in elution buffer according to the manufacturer’s protocol. The 16S rRNA gene of the putative *A*. *gracile* strain from L. Hillestadvannet was amplified using the primers PA and B23s [33, 34], and primers to amplify the *sxt* gene cluster of all Norwegian, German and Spanish strains included in the study were designed using the available PSP toxin biosynthesis gene cluster EU603710 of *Aphanizomenon* sp. NH-5 strain [20]. Primers were designed for sequences covering around 3500 bp each using the FastPCR software [35, 36]. Consecutive sequences were chosen to overlap at 500 nucleotides. After positive amplification intermediate forward and reverse primers were designed with the FastPCR software [35, 36]. The purified 16S rRNA gene was sequenced using the primers as described by Ballot et al. [37] and *sxt* gene cluster fragments were sequenced using the primers depicted in S1 Table. For each PCR product, both strands were sequenced on an ABI 3730 Avant genetic analyzer using the BigDye terminator V.3.1 cycle sequencing kit (Applied Biosystems, Thermo Fisher Scientific Oslo, Norway) according to the manufacturer’s instructions.

### Nucleotide sequence accession numbers

The sequence data were deposited in the European Nucleotide Archive (ENA) under the following accession numbers LT549446 (*A*. *gracile* NIVA-CYA 655 *sxt* gene cluster sequence); LT549447 (*A*. *gracile* UAM529 *sxt* gene cluster sequence); LT549448 (*A*. *gracile* NIVA-CYA 851 *sxt* gene cluster sequence); LT549449 (*A*. *gracile* NIVA-CYA 676 *sxt* gene cluster sequence); LT549450 (*A*. *gracile* NIVA-CYA 851 partial 16S rRNA gene).

### Phylogenetic analysis

A sequence of the 16S rRNA locus in *A*. *gracile* strain NIVA-CYA 851 and sequences of the *sxt* gene cluster in all four *Aphanizomenon* strains were analyzed using the Seqassem software package (version 07/2008) [38]. The Align MS Windows-based manual sequence alignment editor (version 03/2007) [38] was used to obtain DNA sequence alignments, which were then corrected manually. Segments with highly variable and ambiguous regions and gaps making proper alignment impossible were excluded from further analyses. A 16S rRNA gene set containing 1244 positions was used, and *Gloeobacter violaceus* PCC 7421 (AF132790) was employed as an outgroup in the 16S rRNA gene tree. The 16S rRNA sequences from the five *Aphanizomenon* strains and thirty additional Nostocales sequences, derived from GenBank, were included in the 16S rRNA analyses.

A phylogenetic tree for the 16S rRNA gene was constructed using the maximum likelihood (ML) algorithm in Mega v. 6 [39]. The evolutionary substitution model K2+G+I was found to be the best-fitting evolutionary model for the 16S rRNA gene and used for the calculation of the ML tree. ML analyses were performed with 1000 bootstrap replicates using Mega v.6 [39].

Phylogenetic trees based on the complete *sxt* gene cluster and on the *sxtI* gene were also constructed using the ML method in Mega v.6 [39]. The tree for the complete *sxt* gene cluster used *Anabaena circinalis* AWQC131C (EU439557) as an outgroup, whereas *A*. *circinalis* AWQC131C (EU439557), *C*. *raciborskii* T3 (DQ787200) and CENA 303 (JX175233), and *L*. *wollei* (EU603711) were included in the tree for the *sxtI* gene. T92+G was found to be the best fitting model for the complete *sxt* gene cluster and T92 for the *sxtI* gene. These ML analyses were also performed with 1000 bootstrap replicates using Mega v.6 [39].

### Cyanotoxin analysis

#### ELISA

A water sample from Lake Hillestadvannet and *A*. *gracile* strain NIVA-CYA 851 from Hillestadvannet were tested for PSP toxins and cylindospermopsin (CYN) by using Abraxis saxitoxin ELISA and Abraxis cylindrospermopsin ELISA kits (Abraxis LLC, Warminster, PA, USA) following the manufacturer’s instructions. Before analysis, 5 mL of culture material from the field sample and the cyanobacterial strain were frozen and thawed three times to extract the toxins. The ELISA results do not distinguish between dissolved and cell-bound toxins. The color reaction of the ELISA test was evaluated at 450 nm on a Perkin Elmer 1420 Multilabel counter Victor^3^ (Perkin Elmer, Waltham, USA). The analysis for CYN was conducted because in Poland CYN producing *A*. *gracile* strains have been previously described [40].

#### LC-MS analysis

The analytical protocol used in this study for the analysis of PSP toxins in cyanobacterial extracts was conducted following the methods published by Dell'Aversano et al. [41]. 0.5 mL of acetonitrile (containing 0.1% formic acid) was added to 1 mL of NIVA-CYA 851 culture. The mixture was sonicated for 30 min and afterwards filtered through a 0.2 μm membrane and analyzed.

The equipment used for the analysis was a Waters Acquity UPLC coupled to a Sciex 4000QTRAP mass spectrometer. An Ascentis Express OH5 (2.7 μm particle size, 50 x 2.1 mm) column, kept at 20°C, was employed for the chromatographic separation of analytes in HILIC mode. Elution was achieved by a binary gradient of eluents A (1% acetonitrile in water, containing 2 mM ammonium formate and 4 mM formic acid) and B (95% acetonitrile in water, containing 2 mM ammonium formate and 4 mM formic acid) according to the following scheme: t = 0 (90% B), t = 5 min (50% B), t = 7 min (90% B), t = 8 min (90% B). The flow rate was 0.3 mL/min and the total run time was 8 min.

The mass detector was operated in scheduled MRM (Multiple Reaction Monitoring) mode using positive electrospray ionization (ESI+). For toxin identification, two transitions were monitored for each analyte and the most intense transition was then used for quantification.

For PSP toxin identification and quantification, a standard mixture of eight PSP toxins was employed: GTX (gonyautoxin) 1/4 and 5, C (C-toxin) 1/2, STX (Saxitoxin), NeoSTX, decarbamoylSTX, and decarbamoylNeoSTX (NRC-CNRC, Canada). A tentative analysis of other PSP toxins (GTX 2,3,6; 11-OH-STX, decarbamoylGTX 1–4, C 3,4) has also been conducted using detection parameters described in the literature [41]. In S1 Fig representative LC-MS chromatograms for standards and culture extracts are depicted. Due to the difficulty of detecting PSP toxins in real samples because of matrix effects, these quantitative data should be taken as indicative.

## Results

### Morphological and phylogenetic characterisation

**Fig 1:**
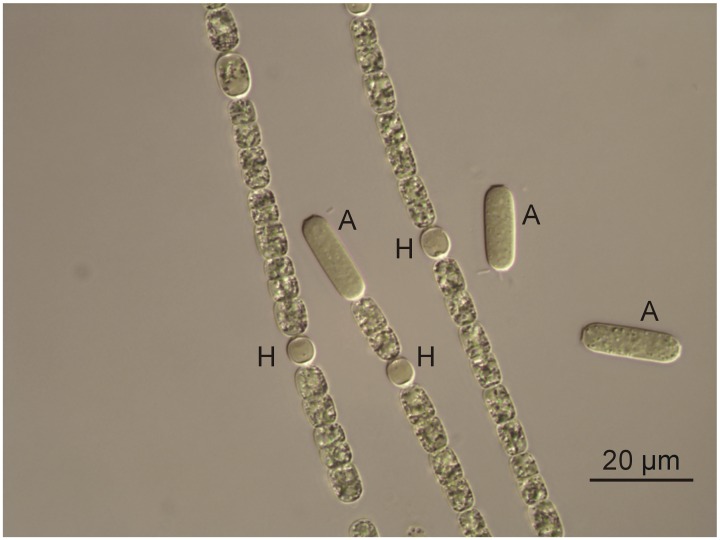
*Aphanizomen gracile* strain NIVA-CYA851 from L. Hillestadvannet, Norway. A = akinetes, H = heterocytes. Scale bar = 20μm.

**Fig 2:**
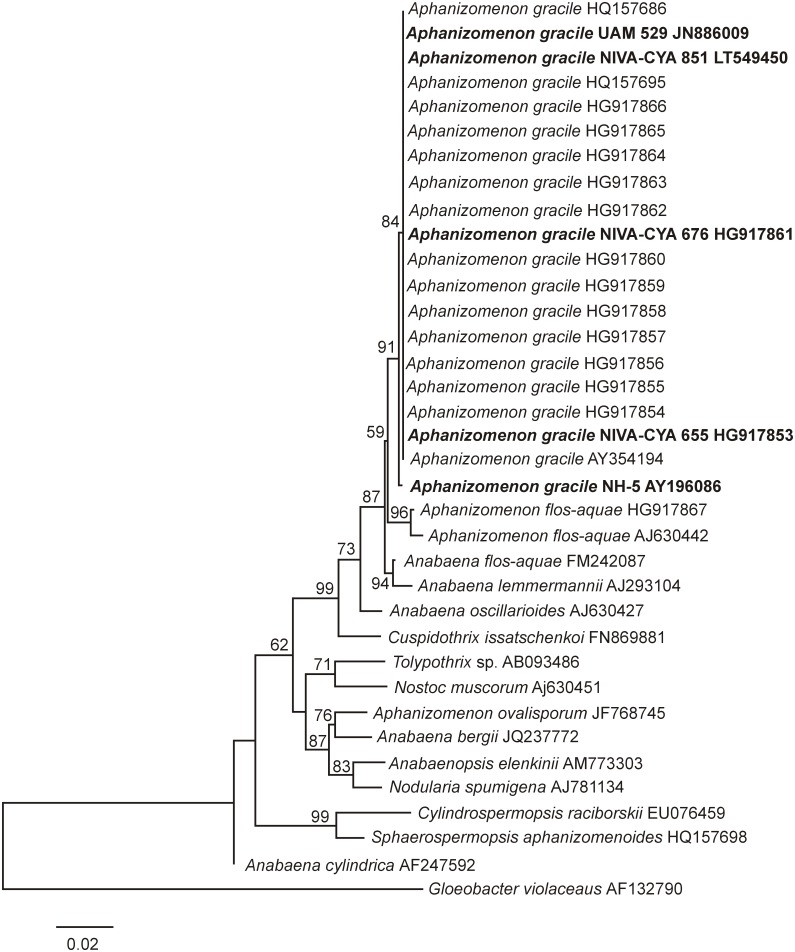
Maximum likelihood tree based on 16S rRNA gene sequences of 36 Nostocales strains. Strains from this study are marked in bold. Bootstrap values above 50 are included. The scale bar indicates 2% sequence divergence.

Based on morphological features and the obtained sequence of the 16S rRNA gene, Norwegian cyanobacterial strain NIVA-CYA 851 was identified as *A*. *gracile* (Fig 1). The filaments were straight or slightly curved and not aggregated in fascicles. The vegetative cells were characterized by a cylindrical to barrel shaped form (min/max/mean length 3.4/10.9/6.6 μm, min/max/mean width 4.2/6.7/5.4 μm); heterocytes were ellipsoidal to spherical (min/max/mean length 5.1/9.1/6.4 μm, min/max/mean width 4.7/6.8/5.7 μm); and the akinetes were characterized by a cylindrical form (min/max/mean length 13.4/36.3/22.5 μm, min/max/mean width 5.3/6.9/6.1 μm). These morphological traits found for strain NIVA-CYA 851 correspond to those described for *A*. *gracile* by Komárek [31]. The phylogenetic relationship of strain NIVA-CYA 851 is presented in the ML tree of the 16S rRNA gene (Fig 2). It is clear that this strain is part of a separate cluster together with the *A*. *gracile* strains from Europe and North America. This cluster is supported by a bootstrap value of 91%.

### PSP toxins and CYN

**Fig 3:**
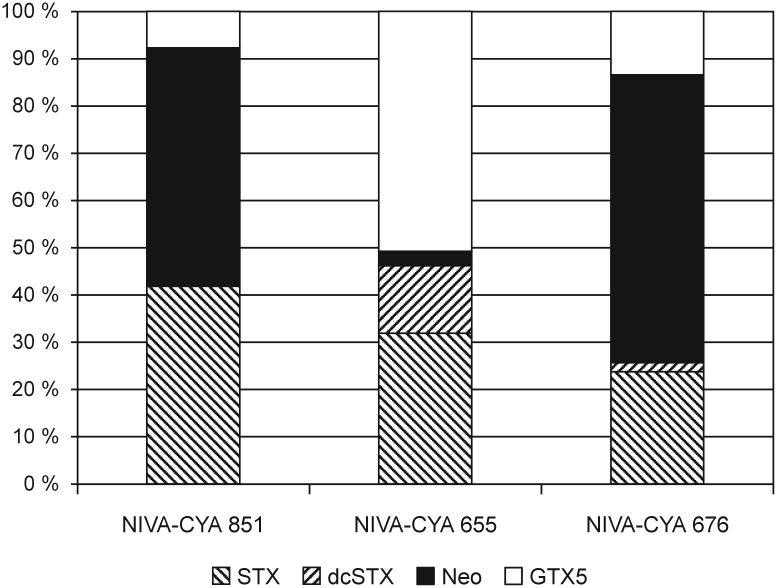
Ratio (%) of PSP toxin variants in *A*. *gracile* strains NIVA-CYA 851 (from Norway) as determined using LC-MS/MS in this study, compared to NIVA CYA 655 and NIVA-CYA 676 (from Germany), analyzed by Ballot et al. [3].

*A*. *gracile* strain NIVA-CYA 851 was confirmed to produce PSP toxins using ELISA. LC-MS measurements confirmed the presence of neosaxitoxin (3695 μg g^-1^ DW), saxitoxin (3064 μg g^-1^ DW), and GTX5 (567 μg g^-1^ DW). In Fig 3 the percentage of each variant is depicted for the Norwegian and German *A*. *gracile* strains NIVA-CYA 851, NIVA-CYA 655 and NIVA-CYA 676. The ratio of PSP toxin variants in *A*. *gracile* strain NIVA-CYA 851 bears greater resemblance to strain NIVA-CYA 676 than to strain NIVA-CYA 655. For the Spanish strain UAM529 the variants STX and dcSTX were found with ESI-LCMS but total PSP toxin concentrations were determined using ELISA only [13]. In the environmental sample from L. Hillestadvannet PSP toxins were not detected by ELISA and have accordingly not been tested with LC-MS. CYN was not detected in the *A*. *gracile* strain NIVA-CYA 851 with ELISA.

**Fig 4:**
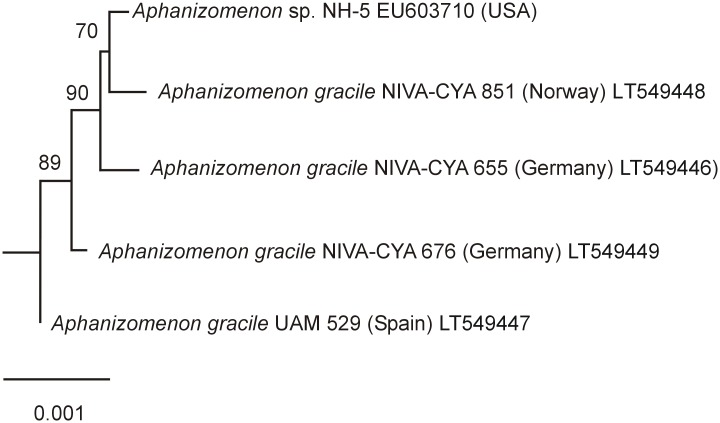
Maximum likelihood tree based on the complete *sxt* gene clusters of five *Aphanizomenon gracile* strains, Outgroup = *Anabaena circinalis* AWQC131C (DQ787201). Bootstrap values above 50 are included. The scale bar indicates 0.1% sequence divergence.

**Fig 5:**
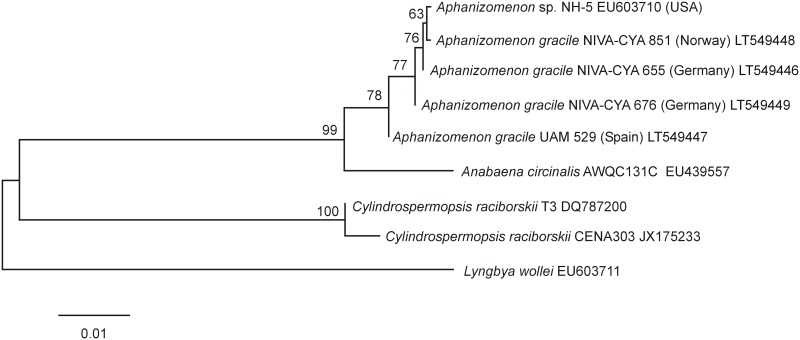
Maximum likelihood tree based on the *sxtI* gene. Bootstrap values above 50 are included. The scale bar indicates 1% sequence divergence.

The complete *sxt* gene cluster sequences of the four Norwegian, German and Spanish *A*. *gracile* strains from this study were aligned with those of reference strain *A*. *gracile* NH-5, and a Maximum Likelihood tree was calculated for phylogenetic investigations. Two separate clusters are clearly distinguished (Fig 4): the *sxt* gene cluster of Norwegian strain NIVA-CYA 851 is most closely related to *A*. *gracile* strain NH-5, and both cluster together with German strain NIVA-CYA 655. Spanish strain UAM 529 is located in one subcluster and the German and Norwegian strains NIVA-CYA 655, 676, & 851 and the reference strain NH-5 form another subcluster (Fig 4). Similar results were found when a ML tree was calculated for the *sxtI* gene only (Fig 5).

In all four investigated *A*. *gracile* strains from Norway, Germany and Spain the *sxt* gene clusters were composed in the same gene order as in reference strain *A*. *gracile* NH-5 [20]. They comprised around 27.3 kb each and altogether 48 variable sites (44 single nucleotide polymorphisms (SNPs) and 4 insertions/deletions (In/Dels) comprising 134 bp, were detected when compared to reference strain *A*. *gracile* NH-5 (Table 1). All four *sxt* gene clusters differed slightly in the number and presence of SNPs and In/Dels. The *sxt* gene clusters of *A*. *gracile* strains NIVA-CYA 851, NIVA-CYA 655, NIVA-CYA 676 and UAM 529 were characterized by the presence of 15, 16, 19 and 23 SNPs, respectively. Of the 44 SNPs detected, 25 SNPs were transversions (Tv) and 14 SNPs were transitions (Tn) (Table 1). In total, 39 SNPs were located in 16 of the 27 coding regions (*sxtPer*, *sxtE*, *sxtW*, *sxtV*, s*xtP*, *sxtR*, *orf24*, *sxtS*, *sxtT*, *sxtU*, *sxtN*, *sxtG*, *sxtH*, *sxtI*, *sxtL* and *orf3*). Nineteen SNPs were synonymous and 20 were non-synonymous substitutions. Five SNPs were located in non-coding regions (Table 1). In coding areas of the *sxt* gene cluster, UAM 529 showed the highest ratio of 1 SNP per 1178 bp, while NIVA-CYA 851 showed the lowest ratio with 1 SNP per 2062 bp. In non-coding areas, 1 SNP per 828 bp was observed in NIVA-CYA 851, while for NIVA-CYA 655 the lowest ratio was 1 SNP per 2487 bp. Only the Norwegian strain NIVA-CYA 851 possessed an insertion of 126 bp in the noncoding area between *sxtA* and *sxtE*, a deletion of one bp in the noncoding region between the *sxtV* and *sxtX* genes and a deletion of 6 nucleotides in the *sxtN* gene. All Norwegian, German and Spanish strains possessed an insertion of one nucleotide T in in the non-coding region between the *sxtX* and *sxtD* genes compared to *A*. *gracile* strain NH-5.

**Table 1:** Genes and non-coding regions in the *sxt* gene cluster with Single Nucleotide polymorphisms compared to *A*. *gracile* strain NH-5. Tn = Transition, Tv = Transversion, In = Insertion, Del = Deletion.

Gene	Tn	Tv	In/ Del	Nonsyn	Total	Strain (SNP, InDel, Non-syn) compared to *A*. *gracile* strain NH-5
*sxtPer*	1			1	1	UAM529 (1, -, 1)
*sxtE*	1			-		NIVA-CYA 655 (1,-,-)
*sxtW*	1			1	1	NIVA-CYA 851 (1,-,-)
*sxtV*	3			?	3	NIVA-CYA 851 (3,-,?); NIVA-CYA 655 (1,-?); NIVA-CYA 676 (1,-,?); UAM 529 (1,-,?);
*sxtP*		1		1	1	NIVA-CYA 851 (1,-,1); NIVA-CYA 655 (1,-,1); NIVA-CYA 676 (1,-,1); UAM 529 (1,-,1)
*sxtR*	2	2		3	4	NIVA-CYA 655 (4,-,3)
*orf24*		2		1	2	NIVA-CYA 851 (1,-,-); NIVA-CYA 655 (2,-,1); NIVA-CYA 676 (1,-,-); UAM 529 (1,-,-);
*sxtS*		1		1	1	NIVA-CYA 655 (1,-,1)
*sxtT*	1			-	1	NIVA-CYA 676 (1,-,-); UAM 529 (1,-,-);
*sxtU*		1		-	1	NIVA 851 (1,-,-)
*sxtN*	1		6	1	2	NIVA-CYA 851 (-,6,-); NIVA-CYA 676 (1,-,1)
*sxtG*	1			-	1	NIVA-CYA 851 (1,-,-)
*sxtH*	3	1		3	4	NIVA-CYA 851 (1,-,1); NIVA-CYA 655 (1,-,1); NIVA-CYA 676 (4,-,3); UAM 529 (1,-,1)
*sxtI*	8	3		4	11	NIVA-CYA 851 (2,-,1); NIVA-CYA 655 (2,-,2); NIVA-CYA 676 (4,-,2) UAM 529 (11,-,4);
*sxtL*	2	2		2	4	NIVA-CYA 655 (2,-2); NIVA-CYA 676 (3,-1); UAM 529 (3,-,1);
*orf3*	1	1		2	2	NIVA-CYA 851 (1,-,1); NIVA-CYA 676 (1,-,1); UAM 529 (1,-,1)
**Subtotal coding**	**25**	**14**	**6**	**20**	**45**	
*sxtA*–*sxtE*	2		126			NIVA-CYA 851 (2,126); NIVA-CYA 676 (1,-); UAM 529 (1,-);
*sxtE*–*sxtW*	1					NIVA-CYA 655 (1,-)
*sxtV*–*sxtX*			1			NIVA-CYA 851(-,1)
*sxtX*–*sxtD*			1			NIVA-CYA 851 (-,1); NIVA-CYA 655 (-,1); NIVA-CYA 676 (-,1) UAM 529 (-,1);
*sxtN—sxtG*	1					NIVA-CYA 851 (1,-)
*sxtL*—*orf3*	1					NIVA-CYA 676 (1,-); UAM 529 (1,-)
**Subtotal noncoding**	**5**	**0**	**128**	**0**	**133**	
**Total**	**30**	**14**	**134**	**20**	**178**	

The highest number of SNPs found was 11 in the *sxtI* gene (UAM529) followed by 4 in *sxtI* and *sxtH* (both NIVA-CYA 676) and 4 in *sxtR* (NIVA-CYA 655) (Table 1).

## Discussion

This is the first study to confirm the presence of PSP toxin producing cyanobacterium *A*. *gracile* in Norway. It is the northernmost occurrence of PSP toxin producing *A*. *gracile* in Europe known so far, and it increases its area of distribution from Western and Central Europe to Northern Europe. From 2004, PSP toxin producing *A*. *gracile* strains have been detected in Portugal, Spain, France and Germany [3, 11–13]. Norwegian strain NIVA-CYA 851 has been clearly identified in this study as *A*. *gracile* using both morphological characteristics and 16S rRNA gene phylogeny. The cluster comprising *A*. *gracile* strains including NIVA-CYA 851 is supported by a bootstrap value of 91%. The assignment of *Aphanizomenon* sp. strain NH-5 to the species *A*. *gracile* suggested by Li et al. [10] and Pereira et al. [11] is also supported by the 16S rRNA phylogeny and our results support the original description of “*atypical*” and “*non-fasciculated*” [18], features more usual for *A*. *gracile* than for *A*. *flos-aquae* [42].

The PSP toxin profile of Norwegian strain NIVA-CYA 851 closely resembles that of German strain NIVA-CYA 676 (AB2008/48) and six other *A*. *gracile* strains isolated from German L. Melangsee due to the dominance of NEO (> 50%) [3]. Although German strain NIVA-CYA 655 (AB2008/16) and 6 other *A*. *gracile* strains isolated from German L. Scharmützelsee have a similar toxin profile, the toxin ratios are dominated by GTX5 (>50%) and are therefore distinct from the strains from L. Melangsee [3]. The lack of dcSTX, however, distinguishes the Norwegian strain from the two German ones [3]. The Spanish strain UAM 529 produces STX and dcSTX [13], and NEO & STX have also been detected in the Portuguese *A*. *gracile* strain LMECYA 40, two French *A*. *gracile* strains PMC 627.10 and PMC 638.10, a Spanish *A*. *gracile* strain UAM 531 and the North American *A*. *gracile* strain NH-5 [11–13, 18]. In a culture of *A*. *gracile* NH-5 three additional putative PSP toxin variants have been observed but not determined [18]. The reasons for the differences in the detected PSP toxin profiles and ratios are still unclear. Varying environmental conditions like changing water hardness or salt concentrations have been shown to have an effect on PSP toxin profiles and ratios in the cyanobacteria *C*. *raciborskii*, *R*. *brookii* or *Cu*. *issatschenkoi* and the eukaryotic dinoflagellate *Alexandrium ostenfeldii* [43–45]. This, however, most likely does not explain the differences in PSP toxin production found in strain NIVA-CYA 851 compared to other *A*. *gracile* strains. The toxin profiles of the two German strains NIVA-CYA 655 and 676 have been analyzed in 2009 but have been grown under similar conditions as NIVA-CYA 851 regarding medium, light and temperature [3].

More likely is an influence on the regulation of the PSP toxin production by the detected SNPs found in 16 genes and orfs of the *sxt* gene cluster in the investigated strains of this study. Especially the non-synonymous SNPs are causing changes in the amino acid composition and probably alter the function of the encoded proteins. Future studies should investigate the role of the SNPs in each of the affected genes.

Another reason for the varying PSP toxin profiles in strains of the same species which possess *sxt* gene clusters with more than 99% sequence similarity can be the varying analytical methodologies applied in the different studies. The use of selected PSP toxin standards only and the varying analytical methods used in different studies could have led to incomplete toxin profiles for the strains investigated.

This study shows that the *sxt* gene clusters from five *A*. *gracile* strains from Norway, Germany, Spain and North America, are highly conserved with similarities of the whole gene clusters exceeding 99.4%. The genes and orfs are arranged in the same order as described from *A*. *gracile* strain NH-5 [20]. Recent studies have confirmed a close relationship of the *sxtA*, *sxtH*, *sxtG*, *sxtI* and *sxtX* genes of the PSP toxin encoding gene cluster of 14 German and two Spanish *A*. *gracile* strains including NIVA-CYA strains 655 & 676, and Spanish strain UAM 529 [3, 13, 29]. Casero et al. [29] have suggested the possibility of certain sub-specific patterns related to geographic location using *sxtH* gene phylogeny. The present study supports the supposed pattern for the German strains NIVA-CYA 655 and NIVA-CYA 676 being separated into two subclusters. However, in contrast to Casero et al. [29], who found a close relationship between German strain NIVA-CYA 655 and Spanish strain UAM 529, the phylogenetic trees of the whole PSP toxin encoding gene clusters and the *sxtI* gene cluster reveal instead that the German and Norwegian strains NIVA-CYA 655, 676, & 851 and the reference strain NH-5 are actually grouped together in one subcluster, while the Spanish strain UAM 529 forms a second subcluster (Fig 4). Interestingly, Norwegian strain NIVA-CYA 851 is closest related to the North American *A*. *gracile* strain NH-5 and NIVA-CYA 655. It is possible that an ancient *A*. *gracile* strain possessing a parent *sxt* gene cluster has evolved into different lines which now co-occurr in the same habitats. Another explanation for the existence of different genotypes could be that the non-synonymous SNPs have had no effect on the function of the *sxt* gene cluster so far.

Mihali et al. [20] have postulated that the PSP toxin biosynthesis evolved in an ancestral cyanobacterium. Pieces of the *sxt* gene cluster are assembled via multiple horizontal gene transfers from a variety of bacterial and cyanobacterial sources and are proposed as the origin of the saxitoxin biosynthetic machinery [46, 47]. Several mechanisms such as losses or rearrangements of genes, as well as recombination, and positive selection have then led to a further evolution of the *sxt* gene cluster [24].

### Insertions and deletions

The insertion of 126 nucleotides found in NIVA-CYA 851 in the noncoding area between *sxtA* and *sxtE* is 69% similar to noncoding regions of the genome of *Nostoc punctiforme* PCC 73102 and plasmid A of *Anabaena variabilis* ATCC 29413 revealed by NCBI Blast. Neither *Nostoc* nor *Anabaena* strains possess the *sxt* gene cluster. The insertion of 126 bp has not been observed in the *sxt* gene clusters of the other three *A*. *gracile* strains of this study or in reference strain *A*. *gracile* NH-5. This suggests that NIVA-CYA 851 could have incorporated this part of the *sxt* gene cluster either via horizontal gene transfer from other cyanobacterial or bacterial sources, or it has previously been lost in the other *A*. *gracile* strains.

The deletion of six nucleotides in the *sxtN* gene in NIVA-CYA851 leads to the loss of the two amino acids lysine and threonine in the encoded sulfotransferase. This deletion has not been observed in the strains from Germany and Spain, or in *A*. *gracile* NH-5. However, it has been observed in the *sxtN* gene in the *sxt* gene cluster of *Anabaena circinalis* (AWQC131C) which is known to produce STX and sulfated and sulfonated PSP toxin variants GTX2/3 and C1/2 [20]. It has been proposed [25] that the protein encoded by *sxtN* is responsible for N-sulfation, leading to the synthesis of C1/2 variants, yet those variants have not been detected in *A*. *gracile* so far. Deletions of 6 nucleotides covering partly the same area (3 nucleotides) have also been observed in the *sxtN* genes of the *sxt* gene cluster of *L*. *wollei* and *C*. *raciborskii* T3, as revealed by NCBI blast.

The insertion of one nucleotide T in the non-coding region between the *sxtX* and *sxtD* genes has been observed in all four Norwegian, German and Spanish strains but not in *Aphanizomenon* sp. strain NH-5. This insertion is located in a non-coding region, but whether it has an effect on toxin production is not yet clear. In strain NIVA-CYA 851 a deletion of a nucleotide T in the non-coding region between the *sxtV* and *sxtX* genes has been observed in this study but, similarly, it is not clear whether this has an effect on PSP toxin production.

### Single nucleotide polymorphisms

Compared to the other *A*. *gracile* strains investigated in this study, NIVA-CYA 851 is characterized by the lowest number of SNPs, while UAM529 has the highest. A single non-coding SNP can be expected—on average—in at least 200–500 base pairs of non-coding DNA, and a single coding SNP in 500–1000 base pairs of coding DNA [48]. The investigated strains in this study are characterized by much lower SNP ratios of between 1 SNP per 1178 bp (UAM529) and 1 SNP per 2062 bp (NIVA-CYA 851) in coding areas, indicating high sequence conservation for the PSP-biosynthesis machinery in *A gracile*. The number of one SNP per 828 bp (NIVA-CYA 851) in non-coding areas of the PSP encoding gene cluster is also much lower than that described by Brumfield et al. [48]. Interestingly, all 39 coding SNPs (89% of all SNPs) are located in 16 of the 27 genes and orfs only. Only eight of these 16 genes and orfs match the core genes described in the known *sxt* gene clusters from *D*. *circinale*, *A*. *gracile* NH-5, *C*. *raciborskii*, *L*. *wollei* and *R*. *brookii* [20, 24].

In this intraspecific study the highest numbers of SNPs (11 in UAM529) has been observed in the *sxtI* gene which suggests it is a suitable marker for further phylogenetic studies on PSP toxin producing *A*. *gracile*. Whether the one (NIVA-CYA 851), two (NIVA-CYA 655 & 676) or four (UAM 529) non-synonymous substitutions cause a functional difference in the encoded product is not yet clear. *SxtI* encodes a carbamoyltransferase [22] and is present in PSP toxin producing cyanobacteria. However, in PSP toxin producing *L*. *wollei* the *sxtI* gene is most likely inactive due to deletions and truncation and only decarbamoylated analogues of saxitoxin are produced by this strain [21, 22]. *SxtI* has also been detected in the non PSP toxin producing *C*. *raciborskii* strain CENA 303 [7], although only a 589 bp long section was investigated by Hoff-Risettii et al. [7]. The *sxtI* gene is therefore most likely not suitable as a selective marker gene for the detection of PSP toxin producers. Casero et al. [29] used a relatively short sequence of the *sxtI* gene (910 of the 1840 nucleotides) to distinguish clear Spanish and German *A*. *gracile* subclusters in a study of 16 *A*. *gracile* strains from Spain and Germany. The use of the whole *sxtI* gene in this study leads to a more variable picture, because parts of the *sxtI* gene not investigated by Casero et al. [29] possess SNPs which lead to a pattern similar to that observed in the phylogenetic tree of the whole PSP toxin encoding gene cluster. These findings suggest that further phylogenetic studies of *A*. *gracile* should make use of the whole *sxtI* gene sequence rather than just part of it. Whether it is a good phylogenetic marker for other cyanobacterial PSP toxin producers such as *D*. *circinalis*, *C*. *raciborskii or R*. *brooki* needs further investigation.

In the Norwegian strain NIVA-CYA 851 the highest numbers of SNPs are found in the *sxtV* gene. The *sxtV* gene is, however, supposed to be inactive in *A*. *gracile* due to a stop codon interrupting the orf [20]. In contrast, in the *C*. *raciborskii* strain T3 the *sxtV* gene is active and seems to be encoding an electron transport system together with *sxtW* [25]. *SxtV* extracts an electron pair from succinate and converts it to fumarate [22]. The encoded product of *sxtW* which is most similar to ferredoxin, transfers the electrons to two ring-hydroxylating dioxygenases encoded by *sxtH* and *sxtT* [20]. In the *sxt* gene clusters of *A*. *circinalis* AWQC131C and *R*. *brookii* D9 *sxtW* and *sxtV* are either not present or truncated and the electron transport is therefore supposed to be complemented by another locus [20, 25]. NIVA-CYA 851 possesses a SNP leading to synonymous substitutions in the *sxtW* gene, which are not found in the other *A*. *gracile* strains.

The only other genes where SNPs are found, in all four strains (*sxtH*, *sxtV*, *sxtP*, and *orf24*), are not as variable as the *sxtI* gene when used for phylogenetic calculations.

Only UAM 529 possesses a non-synonymous SNP in the *sxtPer* gene, which is similar to those in the drug and metabolite transport family [20]. The encoded transporter is most likely responsible for the export of specific PSP toxin variants [20]. *SxtPer* is distinct from *sxtM* which is also most likely involved in the export of PSP toxins out of the cells [20, 25]. No SNPs have been found in the *sxtM* gene of any *A*. *gracile* strain involved in this study.

## Conclusions

This study describes the first detection of PSP toxin producing *A*. *gracile* in Norway. The intraspecific investigation of four *sxt* gene clusters in toxin producing *A*. *gracile* strains from Norway, Germany and Spain, together with a fifth reference strain of *A*. *gracile* NH-5 from the USA (spanning latitudes between 40° and 59° north), has shown that gene composition is highly conserved within *A*. *gracile*. However, the variability in the numbers and positions of both SNPs and insertions & deletions in the *sxt* gene clusters highlights important differences between strains. In some locations, such as in Germany, several strains coexist, but elsewhere they seem geographically distinct e.g. in Norway, Spain and the USA. Further studies encompassing a wider geographic area will be necessary to determine the precise distribution of the strains.

The highest genetic variability has been observed in the *sxtI* gene, which expresses both inter- and intra-specific patterns. It is therefore recommended for further phylogenetic research of PSP toxin producing *A*. *gracile*. The suitability of the *sxtI* gene as a good phylogenetic marker for other cyanobacterial PSP toxin producers such as *D*. *circinalis*, *C*. *raciborskii or R*. *brooki* needs to be further investigated.

## Supporting Information

S1 Fig Chromatograms of a) *A*. *gracile* strain NIVA-CYA 851 and b) of a mixture of PSP toxin standards. In both chromatograms, the most intense transition is reported per each analyte. For clarity, the transitions have been stacked on *y* axis in the chromatogram depicting the mixture of standards.
(PDF)

S1 Table Primers for PCR and sequencing of the *sxt* gene cluster of *Aphanizomenon gracile*. (PDF)
