# Supplementary material for: Variability in the sxt Gene Clusters of PSP Toxin Producing Aphanizomenon gracile Strains from Norway, Spain, Germany and North America
Source: PLoS One. 2016 Dec 1;11(12):e0167552. doi: 10.1371/journal.pone.0167552 (PMC5132012; doi:10.1371/journal.pone.0167552)

S1 Fig. Chromatograms of a) *A. gracile* strain NIVA-CYA 851 and b) of a mixture of PSP toxin standards. In both chromatograms, the most intense transition is reported per each analyte. For clarity, the transitions have been stacked on *y* axis in the chromatogram depicting the mixture of standards.

a

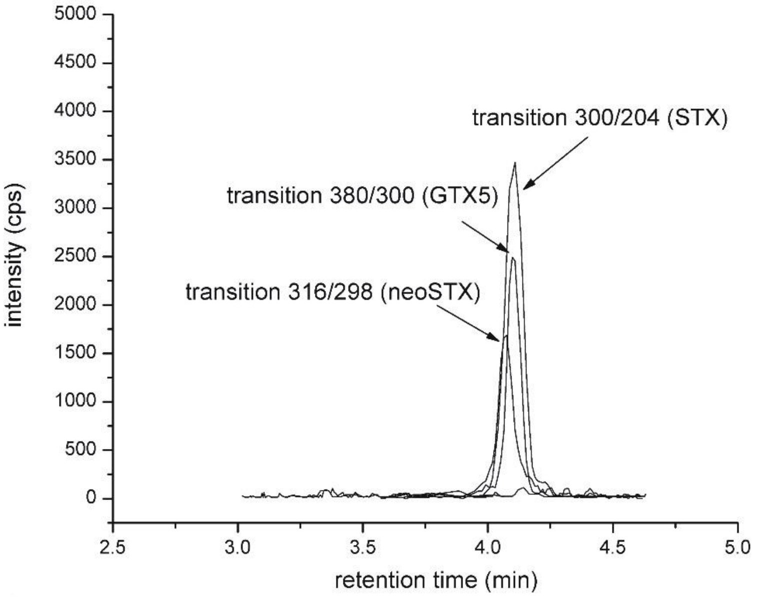

b

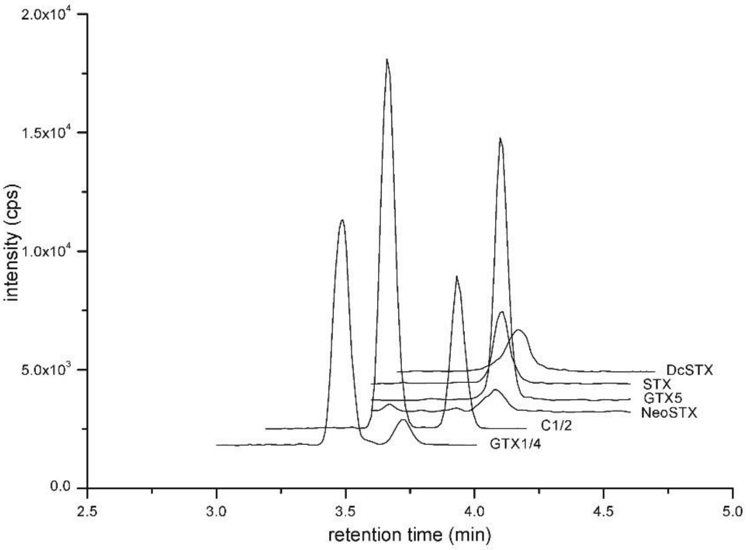

Supplement: S1 Fig — In both chromatograms, the most intense transition is reported per each analyte. For clarity, the transitions have been stacked on y axis in the chromatogram depicting the mixture of standards. (PDF) [file pone.0167552.s001.pdf]
