# Supplementary material for: Variability in the sxt Gene Clusters of PSP Toxin Producing Aphanizomenon gracile Strains from Norway, Spain, Germany and North America
Source: PLoS One. 2016 Dec 1;11(12):e0167552. doi: 10.1371/journal.pone.0167552 (PMC5132012; doi:10.1371/journal.pone.0167552)
Supplement: S1 Table — (PDF) [file pone.0167552.s002.pdf]

S1 Table Primers for PCR and sequencing of the *sxt* gene cluster of *Aphanizomenon gracile*

Segment	Primer	Use	primer sequence	genes covered
1	sxt32f	PCR, sequencing	aaaacccggacgagccgggta	*psbh* partial, *sxtPer*, *orf1*, *sxtC* *orf2*, *sxtB* partial
	sxt837f	sequencing	aatttccaggctaaagcagc	
	sxt1614f	sequencing	gaatgcccctcggttggcag	
	sxt1900r	sequencing	gacaacatctgccccagaa	
	sxt2532r	sequencing	ttcgccagcctccaaaacga	
	sxt3205r	PCR, sequencing	tgaggtacgcccctgcaactc	
2	sxt2532f	PCR, sequencing	tcgttttggaggctggcgaa	*sxtC*, *orf2*, *sxtB* partial
	sxt3289f	sequencing	gcatgaacggaaatccgtc	
	sxt3155r	sequencing	acaagagatgccagtggtg	
	sxt3700r	PCR, sequencing	ccgatggaatctgctctgacc	
3	sxt3289f	PCR, sequencing	gcatgaacggaaatccgtc	*sxtB* partial, *sxtA* partial
	sxt3908f	sequencing	agatttggcggtccaaagg	
	sxt4533f	sequencing	ttcggtattggcagccgta	
	sxt5269f	sequencing	gccagacagcacgcttcat	
	sxt5518r	sequencing	ggaagcgattccaccggct	
	sxt4881r	sequencing	aacttccgcaacgcttgga	
	sxt4245r	sequencing	cgatcgctcttggtaaacca	
	sxt3700r	sequencing	ccgatggaatctgctctgacc	
	sxt5572r	PCR, sequencing	atgggttcccaacgggaagc	
4	sxt5516f	PCR, sequencing	tggatgcaagtcaagacct	*sxtA* partial, *sxtE, sxtW* partial
	sxt6206f	sequencing	ctccgtcatcggcatttggga	
	sxt6876f	sequencing	ccgtgcttagtgaccactcg	
	sxt77550f	sequencing	tacggcggtattcaaggctcg	
	sxt8207f	sequencing	cgttttgctagagggtcaa	
	sxt6091r	sequencing	gagaatttcggcatgggta	
	sxt6800r	sequencing	tttcgatgcgtttcaggga	
	sxt7511r	sequencing	tgtactgtttggcgattgtc	

	sxt8224r	sequencing	tgaccctctagcaaaacga	
	sxt8998r	PCR, sequencing	gcaatctcatgccaatgttgc	
5	sxt8533f	PCR, sequencing	tagtcagctttggcaatgc	*sxtW, sxtV* partial
	sxt9203f	sequencing	atcacgtctcacccaaagcct	
	sxt9206r	sequencing	aaggctttgggtgagacgt	
	sxt9878r	PCR, sequencing	aaaagctggcattgaccct	
6	sxt9203f	PCR, sequencing	atcacgtctcacccaaagcct	*sxtV* partial, *sxtX* ,
	sxt9954f	sequencing	gcataaacgggagctttcag	
	sxt10720f	sequencing	ccaattaccagcacgtcag	
	sxt11445f	sequencing	tgacaaatcgcggttttcca	
	sxt11254r	sequencing	ccctactcgacgagaaagc	
	sxt10603r	sequencing	gcatcatggcaccttctcc	
	sxt9878r	sequencing	aaaagctggcattgaccct	
	sxt11727r	PCR, sequencing	agtaattcgcctcttgtgg	
7	sxt11505f	PCR, sequencing	tgacaaatcgcggttttcca	*sxtX* partial, *sxtD* , *sxt* P partial
	sxt12246f	sequencing	tttccgctcagccaccacgat	
	sxt12239r	sequencing	tagctttactggcggactt	
	sxt12938r	PCR, sequencing	tactagcagcccatttcc	
8	sxt12845f	PCR, sequencing	ccgttaagttggatgcaggag	*sxtP* partial, *sxtQ* , *sxtR*
	sxt13603f	sequencing	cttgacctagccgtgacaa	
	sxt14298f	sequencing	tgaaacaatgcgcgatcct	
	sxt15199f	sequencing	tttagcatgggaatgccct	
	sxt15198r	sequencing	gagacacagtttccttgcgct	
	sxt14344r	sequencing	cgatagttccccagaagca	
	sxt15722r	PCR, sequencing	tatttcctcgcaatcccga	
9	sxt14005f	PCR, sequencing	tttcccacctgaaccaacggt	*sxtQ* partial, *sxtR* , *orf24* , *sxtS, sxtT* partial
	sxt14344f	sequencing	cgatagttccccagaagca	
	sxt15199f	sequencing	tttagcatgggaatgccct	

	sxt15825f	sequencing	ctatggcacaccgcatctgct	
	sxt16482f	sequencing	atggtgagacgatccacta	
	17135f	sequencing	caccaactgtctggcacac	
	sxt17129r	sequencing	aacattgcggctcctccagga	
	sxt16501r	sequencing	ttagtggatcgtctcacca	
	sxt15722r	sequencing	tatttcctcgcaatcccga	
	sxt15198r	sequencing	gagacacagtttccttgcgct	
	sxt17820r	PCR, sequencing	tcacacgaaacgcactagctt	
10	sxt17523f	PCR, sequencing	caccgaggtgtggctctgtc	*sxtT* partial, *sxtU, sxtN,*
	sxt18213f	sequencing	cggatcctacatgcccaacag	
	sxt18842f	sequencing	actggagacgagcattcga	
	sxt19424f	sequencing	gatgagcctctattcgcccca	
	sxt19440r	sequencing	gcgaatagaggctcatcat	
	sxt18702r	sequencing	aggtaatgcctgaccgccac	
	sxt18144r	sequencing	tttgagatccgttccacgt	
	sxt20246r	PCR, sequencing	ggagtttacacggctggat	
11	sxt19424f	PCR, sequencing	gatgagcctctattcgcccca	*sxtN* partial, *sxtG* partial
	sxt20172f	sequencing	cataaccgttcagacaggga	
	sxt20512	sequencing	ccctatccaccggagatgat	
	sxt20246r	sequencing	ggagtttacacggctggat	
	sxt21104r	PCR, sequencing	attttgccgggagcaagag	
12	sxt20512f	PCR, sequencing	ccctatccaccggagatgat	*sxtG* partial, *sxtH, sxtM*
	sxt21170f	sequencing	tggttgcaccttaccccaa	
	sxt21642f	sequencing	aagaccactgtccccaccgagg	
	sxt22253f	sequencing	ttgatcacgtggaactgccct	
	sxt22943f	sequencing	gctgttaggccaacaagaa	
	sxt23157f	sequencing	aaaccgttgggtgcaagaa	
	sxt21104r	sequencing	attttgccgggagcaagag	
	sxt21727r	sequencing	tctccatccgtgataagca	
	sxt22455r	sequencing	ctgtgcagcgatctgatggcac	

	sxt22830r	sequencing	caaaagcctcggctgcttgtg	
	sxt23439r	sequencing	ctgtttgggctgcaatctc	
	sxt23995r	PCR, sequencing	aacaccttttcggcgggta	
13	sxt23157f	PCR, sequencing	aaaccgttgggtgcaagaa	*sxtM* partial, *sxtI*, *sxtJ*, *sxtK*, *sxtL* partial
	sxt24212r	sequencing	aagtttgagcgattgctgg	
	sxt24861f	sequencing	tggtactgcgtttggcgaa	
	sxt25495f	sequencing	gcagctatttgggatcgagaa	
	sxt26191f	sequencing	gctccaatgatgcgaagct	
	sxt24260r	sequencing	atccatttggcgcataggcta	
	sxt25083r	sequencing	gctgttggaatgcggggtt	
	sxt25651r	sequencing	agcacaaccagttcgtgcct	
	sxt6387r	sequencing	ccccaaggataccagtgtgaa	
	sxt26996r	PCR, sequencing	tgactcaaagcacgggagtt	
14	sxt26522f	PCR, sequencing	tctttggagtggtgttcgt	*sxtL* partial, *orf3*, *ubiA* partial
	sxt27204f	sequencing	ggggttttctgctcaagca	
	sxt27924f	sequencing	acatcaattgcgactccat	
	sxt28467f	sequencing	ttgggttgcatctagctgg	
	sxt29091f	sequencing	gctacggacaaccagaagc	
	sxt26996r	sequencing	tgactcaaagcacgggagtt	
	sxt27422r	sequencing	tcgcgttgaaatggaccgcca	
	sxt28083r	sequencing	atcaacccaacggcgtacc	
	sxt28790r	sequencing	tctcctagctggagtaggt	
	sxt29422r	PCR, sequencing	atccggctgttaaggtgga	
